# Microbial community in microbial fuel cell (MFC) medium and effluent enriched with purple photosynthetic bacterium (*Rhodopseudomonas sp.*)

**DOI:** 10.1186/s13568-014-0022-2

**Published:** 2014-04-01

**Authors:** Tae-Jin Park, Weijun Ding, Shaoan Cheng, Manreetpal Singh Brar, Angel Po Yee Ma, Hein Min Tun, Frederick C Leung

**Affiliations:** 1 School of Biological Sciences, The University of Hong Kong, Hong Kong, PR China; 2 State Key Laboratory of Clean Energy Utilization, Department of Energy Engineering, Zhejiang University, Hangzhou 310027, PR China; 3 Bioinformatics Center, Nanjing Agriculture University, Nanjing, PR China

**Keywords:** Purple color characteristic of Rhodopseudomonas, Microbial community, Hydrogen oxidizing bacteria, Hydrogenotrophic methanogens, Syntrophic relationship

## Abstract

High power densities have been obtained from MFC reactors having a purple color characteristic of *Rhodopseudomonas*. We investigated the microbial community structure and population in developed purple MFC medium (DPMM) and MFC effluent (DPME) using 16S rRNA pyrosequencing. In DPMM, dominant bacteria were *Comamonas* (44.6%), *Rhodopseudomonas* (19.5%) and *Pseudomonas* (17.2%). The bacterial community of DPME mainly consisted of bacteria related to *Rhodopseudomonas* (72.2%). Hydrogen oxidizing bacteria were identified in both purple-colored samples: *Hydrogenophaga* and *Sphaerochaeta* in the DPMM, and *Arcobacter*, *unclassified Ignavibacteriaceae*, *Acinetobacter, Desulfovibrio* and *Wolinella* in the DPME. The methanogenic community of both purple-colored samples was dominated by hydrogenotrophic methanogens including *Methanobacterium, Methanobrevibacter* and *Methanocorpusculum* with significantly lower numbers of *Methanosarcina*. These results suggeste that hydrogen is actively produced by *Rhodopseudomonas* that leads to the dominance of hydrogen consuming microorganisms in both purple-colored samples. The syntrophic relationship between *Rhodopseudomonas* and hydrogenotrophic microbes might be important for producing high power density in the acetate-fed MFC under light conditions.

## Introduction

Microbial fuel cell (MFC) is a new technology in renewable energy. It generates electrical power while accomplishing waste water treatment by utilizing microorganisms (Pant et al. [2012]). Although MFCs have been comprehensively investigated, this technology is still at an early stage and under extensive laboratory research. Over the past 10 years, considerable effort has been made to improving power generation efficiency, focusing mainly on different MFC setups and new materials (Kim et al. [2008]; Lovley [2006]; Sleutels et al. [2012]). Microbial ecology studies in MFC systems are important for understanding the mechanism of microbial electricity generation (Rabaey et al. [2007]; Rabaey and Rozendal [2010]). In addition, MFC systems also provide insight into the physiological roles of microbes and better understanding of interactions in complex microbial communities within natural environments (Bretschger et al. [2010]).

The bacterial population and predominant species vary depending on operational conditions such as inocula, substrate nature and electrode materials (Sun et al. [2012]; Logan and Regan [2006]; Logan [2009]). In general, MFCs using mixed culture produce more power than ones with pure culture (Watson and Logan [2010]). However, complex syntrophic interactions existing in MFC systems relating to high power densities have not been well studied. Previous studies have also shown that MFC performances are affected by light, which can cause the solution medium of respective MFCs to enrich in the phototrophic purple nonsulfur (PNS) bacterium, *Rhodopseudomonas palustris* DX-1. This bacterium has been shown to produce higher power densities in pure culture than in mixed culture and increase power production along with light intensity. In contrast to, the *R. palustris* ATCC 17001 does not generate power (Xing et al. [2008]). The genus *Rhodopseudomonas* liberates hydrogen when illuminated anaerobically in the presence of a carbon source such as acetate and malate (Barbosa et al. [2001]). The hydrogen production is mediated by a nitrogenase enzyme and dependent on light (Basak and Das [2007]; Rey et al. [2007]).

Previous MFC microbial ecology studies have used Sanger-based 16S rRNA sequencing for microbial community characterization. Recent advances in next generation sequencing such as pyrosequencing offer a better alternative for comprehensively characterizing microbial communities, especially in terms of less abundant members (Dowd et al. [2008]; Huse et al. [2007]; Lee et al. [2010]). 454 pyrosequencing of 16S rRNA gene has been revealed highly diverse microbial communities in MFCs and MECs (Lee et al. [2010]; Jia et al. [2013]; Lu et al. [2012a]). The present study aimed at better understanding the high power density achieved in MFC reactors having purple color characteristic of *Rhodopseudomonas.* To do this, the microbial communities in the purple-colored samples collected from a media bottle and a MFC reactor respectively were investigated using 16S rRNA amplicon pyrosequencing.

## Materials and methods

### Sample source

The MFC medium was kept in a common media bottle (320 mL) sealed with its cap and placed under natural sunlight and fluorescent lamp lighting (around 400 lx) without any inoculum. Four weeks later the developed purple medium was collected. It was observed that coloring of the medium serendipitously became purple over time. The naturally developed purple MFC effluent was collected from a single-chamber air-cathode MFC (4.5 L) having four homemade brush anodes (60 mm in diameter and 150 mm in length) inoculated with the primary clarifier overflow of a local wastewater treatment plant. The setup has been operated in batch mode for approximately two years using acetate (1.0 g/L) as a substrate at 30°C. Both media had the following composition: 1.0 g/L sodium acetate in 50 mM phosphate buffer solution (PBS, pH 7.0) containing (per liter deionized water): KCl, 0.13 g/L; NaH_2_PO_4_ · 2H_2_O, 2.75 g/L; Na2HPO4 · 12H_2_O, 11.466 g/L; NH _4_Cl, 0.31 g/ L, metal (12.5 mL/L) and vitamins (5 mL/L) (Lovley and Phillips [1988]). For simplicity, the MFC media bottle medium will now be referred to as “developed purple MFC medium (DPMM)” and the MFC reactor effluent as “developed purple MFC effluent (DPME)”.

### Total DNA extraction and 16S rRNA gene pyrosequencing

Each purple-colored sample (5 ml) was centrifuged at 5630× g for 10 minutes to collect microbial biomass. Total genomic DNA was extracted using a PowerSoil DNA isolation kit (MO-BIO) according to the manufacturer’s protocol. Amplicon libraries were constructed for 454 sequencing using different sets of primers: 27 F (5′GAGTTTGATCMTGGCTCAG-3′) and 518R (5′-WTTACCGCGGCTGCTGG-3′) targeting the bacterial domain, 519 F (5′-CAGCMGCCGCGGTAA −3′) and 915R (5′-GTGCTCCCCCGCCAATTCCT −3′) targeting the archaeal domain (Baker et al. [2003]). The forward primers contained a 10 base barcode sequence positioned between the adapter and the primer sequence. The barcode sequences were unique for each sample.

The amplification of 16S rDNA was performed in a final volume of 50 μl containing 5 μl of 10X buffer, 1 μl of dNTP mixture (200 mM), 0.5 μl of (20XU) FastStart Taq DNA Polymerase (Roche), 1 μl of each primer (10 μM), 1 μL of DNA template (50 ng/μL) and water. The PCR conditions were as follows: 94°C for 3 min; 30 cycles of 94°C for 45 S, 55°C for 45S, followed by 72°C for 1 min; and a final extension at 72°C for 10 min. The PCR products were gel purified on 1% agarose gels using a Quick Gel Extraction Kit (Invitrogen). The purified 16 s amplicons were quantified with Picogreen and pooled in equal concentrations prior to emulsion PCR. Amplicon pyrosequencing was performed using a 454/Roche GS Junior instrument (454 Life Sciences).

### Sequence analysis

The *sffinfo* command was used to extract fasta format (sequence data) and quality (quality scores) files from a raw.sff file with –s and –q options, respectively. The following steps were carried out by the QIIME (Quantitative Insights into Microbial Ecology) software package (Caporaso et al. [2011]). Output fasta and quality files were filtered to exclude sequences with lengths (<150 bp) and quality score (<50). Raw sequences with uncorrectable barcodes were removed. The remaining sequences were sorted by a mapping file to their relevant samples according to the barcode. The denoised dataset was clustered into operational taxonomic units (OTUs) with 97% similarity threshold, using the UCLUST algorithm. The representative sequences were selected and aligned with greengenes core set of aligned sequences using Pynast. Taxonomy was assigned using the greengenes database. For OTUs that represented more than 5% of the sequences for any sample, taxonomic identification at the genus level was determined using basic local alignment search tool (BLAST) to compare the representative sequence for that OTU against the National Center for Biotechnology Information (NCBI) nucleotide non-redundant database. Phylogenetic trees were constructed using the software MEGA v5 with the neighbor joining criterion, and 1,000 times of bootstrap resampling was performed to assess the confidence of tree topologies (Kumar et al. [2008]).

## Results

### Bacterial community revealed on the V1-V3 region of the 16S rRNA gene

**Figure 1:**
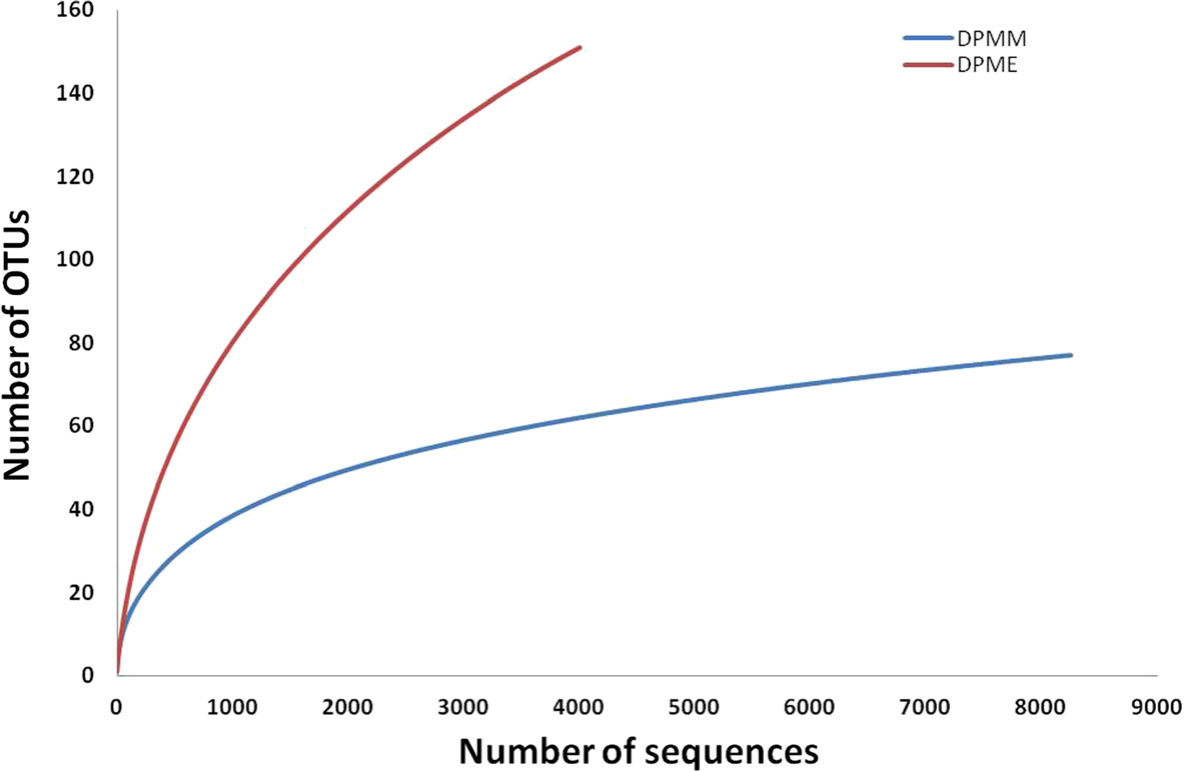
Rarefaction curves for bacterial 16S rRNA gene sequences with cut-off threshold of 97% similarity in DPMM and DPME.

**Figure 2:**
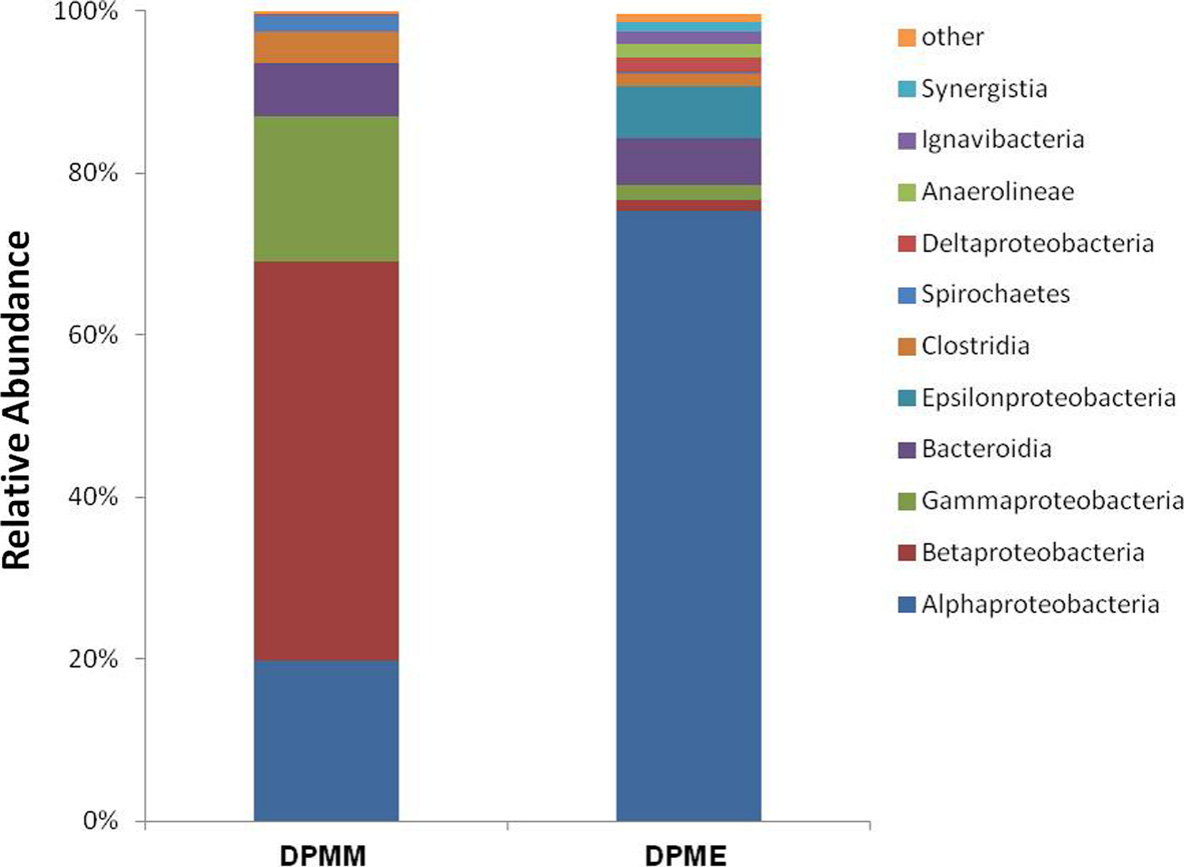
Relative abundances of bacterial phyla and proteobacterial classes in DPMM and DPME.

The bacterial communities from DPMM and DPME were revealed using the 16 s rRNA primer set (V1- V3). The number of total reads was 8253 reads in DPMM and 4008 reads in DPME. We obtained 77 and 151 operational taxonomic units (OTUs) from DPMM and DPME at 97% similarity respectively (Figure 1). The rarefaction curve of the DPMM tends to reach a plateau, but the DPME showed that new bacterial phylotypes continued to emerge even after 4,000 reads. *Betaproteobacteria* (49.3%), *Alphaproteobacteria* (19.8%) and *Gammaproteobacteria* (17.8%) were major bacterial phyla in DPMM. On the other hand the DPME primarily had *Alphaproteobacteria* (75.4%), *Epsilonproteobacteria* (6.5%) and *Bacteroidetes* (5.7%) phyla (Figure 2).

**Figure 3:**
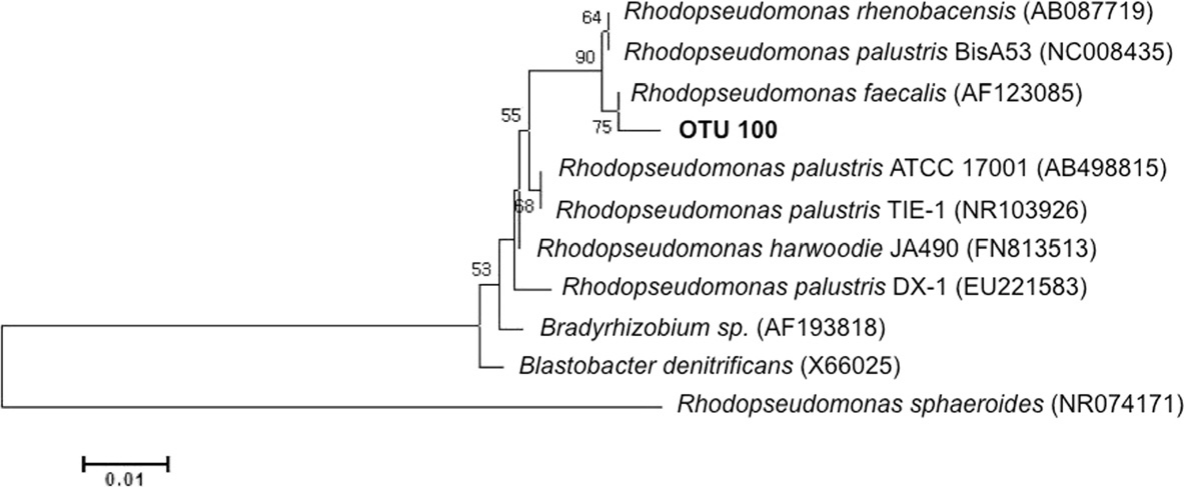
**Phylogenetic tree based on 16S rRNA gene sequences showing the relationships of OUT 100 with members of the genus*Rhodopseudomonas*.** Bootstrap values (>50%) are shown on the nodes from 1000 bootstrap replicates. The scale bar represents 5% difference in nucleotide sequences.

The community of the DPMM consisted mainly of bacteria related to the genera *Comamonas* (42.8%), *Rhodopseudomonas* (19.6%) and *Pseudomonas* (17.1%). The genus *Rhodopseudomonas* (73.3%) was the most dominant followed by *Arcobacter* (5.25%) in the DPME (Table 1). The phylogenetic analysis based on sequences of partial 16S rRNA genes indicated that *Rhodopseudomonas* was closely related to *Rhodopseudomonas faecalis* (AF123085) with 99% similarity (Figure 3). However, the predominant bacteria in DPMM, *Comamonas* and *Pseudomonas*, were found with low abundance in DPME accounting for 0.47% and 0.05% respectively. *Comamonas* and *Arcobacter* had a high similarity of 99% to *Comamonas testosteroni* (NR102841) and *Arcobacter butzleri* ED-1 (NR074567), respectively. Both are known exoelectrogenic bacteria in an acetate-fed MFC (Fedorovich et al. [2009]; Juang et al. [2011]). *Hydrogenophaga* (3.99%) and *Sphaerochaeta* (1.88%) were found at higher abundance in the DPMM compared to the DPME (Table 1). It has been reported that the genus *Hydrogenophaga* were highly abundant in acetate-fed MFC and some *Hydrogenophaga* species showed hydrogen-dependent power generation (Kimura and Okabe [2013b]). The genus *Sphaerochaeta* was detected in the hydrogen-producing MECs (Lu et al. [2012b]). Higher abundance of the unclassified *Ignavibacteriaceae* (1.66%), *Acinetobacter* (0.68%), *Desulfovibrio* (0.8%), *Wolinella* (1.01%) and *Azospirillum* (1.14%) were present in DPMM than in DPME; these genera were commonly found in electrode biofilm community for MFCs and MECs (Pisciotta et al. [2012]; Liu et al. [2012]; Yu et al. [2012]). These bacteria are known to be able to use hydrogen as an electron donor (Wong et al. [1986]; Gross and Simon [2003]; Yong et al. [2002]; Iino et al. [2010]; Yuan et al. [2012]). *Geobacter* sp*.*, a known exoelectrogenic bacteria, was found to account for 0.4% abundance in DPME, but was negligible in the DPMM.

**Table 1:** Relative abundances of bacterial OTUs based on the V1-V3 primer set (>0.1% of total population) in DPMM and DPME

**Classification**	**DPMM (%)**	**DPME (%)**	**OTUs**	**Genbank closest match (accession no. and similarity)**
*Rhodopseudomonas*(*Alphaproteobacteria*)	19.63	73.28	100	*Rhodopseudomonas faecalis* (JX282402, 99%)
*Comamonas*	42.82	0.47	27	*Comamonas testosteroni* (NR102841, 99%)
*Pseudomonas*	17.12	0.05	148	*Pseudomonas stutzeri* (KF171338, 99%)
Unclassfied *Bacteroidales*	3.27	0.23	25	Uncultured bacterium (JQ724350, 99%)
*Arcobacter*	0	5.25	66	*Arcobacter butzleri* ED-1 (NR074567 99%)
Unclassfied *Porphyromonadaceae*	1.48	0.29	7	Uncultured bacterium (JF568463, 99%)
*Hydrogenophaga*	3.99	0.05	153	*Hydrogenophaga* sp. (AB746948, 98%)
Unclassfied *Clostridiales*	1.46	0.23	137	Uncultured *Anaerovorax* sp. (JQ087107, 98%)
*Sphaerochaeta*	1.88	0.13	127	Uncultured bacterium (JQ245575, 99%)
*Azospirillum*(*Alphaproteobacteria*)	0	1.14	73	Uncultured bacterium (GQ152964, 99%)
Unclassfied *Methylocystaceae*(*Alphaproteobacteria*)	0	1.38	135	Uncultured *Rhizobiales* (JQ723649, 99%)
*Acinetobacter*(*Gammaproteobacteria*)	0.3	0.68	136	Uncultured bacterium (KC001216, 99%)
*Geobacter*(*Deltaproteobacteria*)	0.01	0.23	190	Uncultured *Geobacter* (JQ724335, 99%)
*Desulfovibrio*(*Deltaproteobacteria*)	0.1	0.8	122	Bacterium enrichment (GU196246, 98%)
Unclassfied *Desulfobacteraceae*(*Deltaproteobacteria*)	0	0.31	140	Uncultured bacterium (GQ152952, 98%)
*Wolinella*(*Epsilonproteobacteria*)	0	1.01	29	Uncultured bacterium (JQ987976, 100%)
*Clostridium*(*Firmicutes*)	0.33	0	79	Iron-reducing bacterium (FJ802349, 97%)
*Bacteroides*(*Firmicutes*)	0.51	0	46	Uncultured bacterium (JQ983412, 97%)
*Oscillospira*(*Firmicutes*)	0.59	0	168	Uncultured *Oscillibacter* (JX462523, 99%)
Unclassfied *Catabacteriaceae*(*Firmicutes*)	0	0.36	9	Uncultured bacterium (KC408589, 99%)
Unclassfied *Ruminococcaceae*(*Firmicutes*)	0.22	0.1	125	Uncultured bacterium (JQ983937, 99%)
*Dysgonomonas*(*Bacteroidetes*)	0.4	0.1	156	Uncultured *Dysgonomonas* (JX462548, 99%)
Unclassfied *Porphyromonadaceae*(*Bacteroidetes*)	0	1.4	113	Uncultured bacterium (JN792255, 99%)
WCHB1-05 (*Chloroflexi*)	0	0.26	141	Uncultured bacterium (JX224526, 99%)
WCHB1-50 (*Chloroflexi*)	0	1.01	191	Uncultured bacterium (AJ387902, 96%)
Unclassfied *Ignavibacteriaceae* (*Chlorobi*)	0	1.66	54	Uncultured *Ignavibacterium* (JQ724356, 100%)
*Aminiphilus* (*Synergistetes*)	0	1.01	75	Uncultured bacterium (KC736302, 99%)

### Archaeal and bacterial communities revealed by the V3-V5 region of the 16S rRNA gene

**Figure 4:**
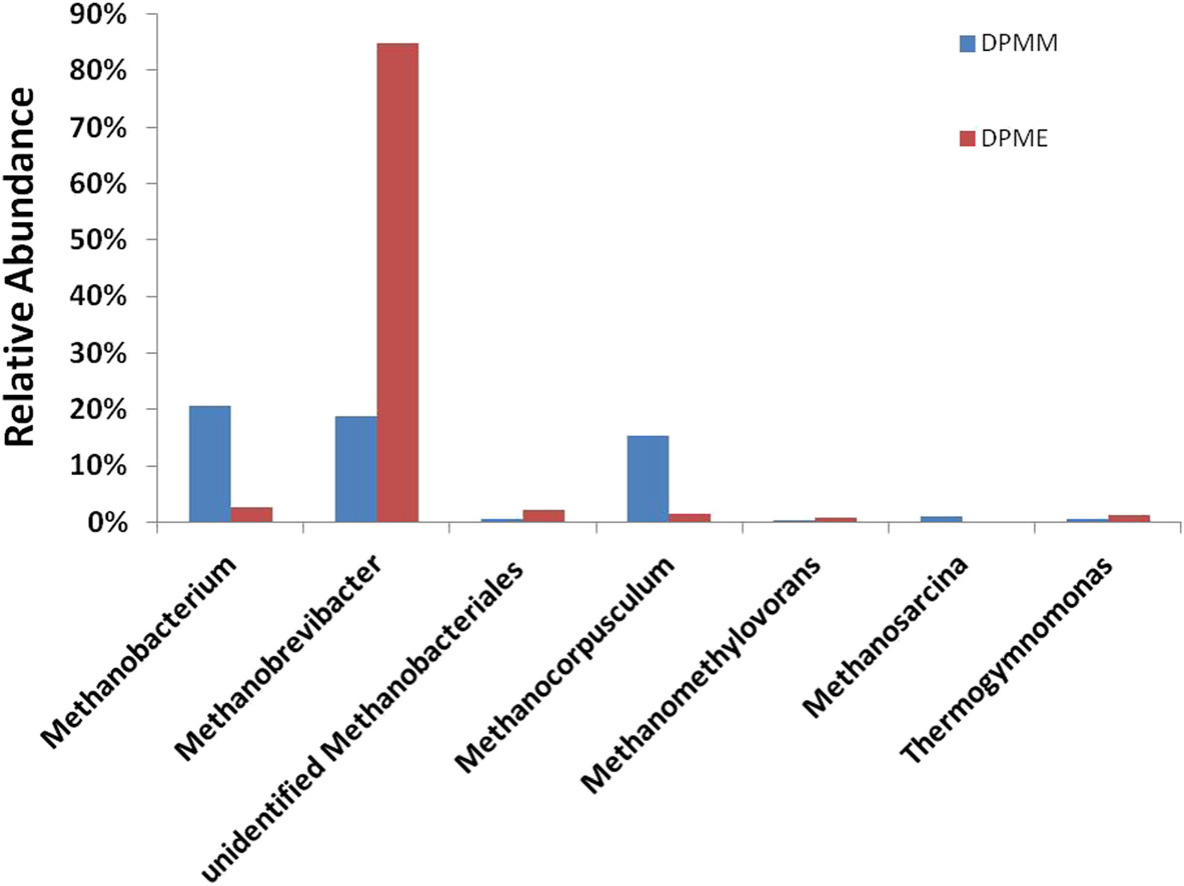
Relative abundances of archaeal genera in DPMM and DPME.

The number of total high quality sequences obtained from raw pyrosequencing data was 4586 reads. We obtained 59 OTUs for DPMM and 38 OTUs for DPME at 97% similarity. The rarefaction curves were far from the plateauing due to the limitation in sequencing depth for covering a much higher bacterial diversity than archaeal diversity (Additional file 1: Figure S1). The dominance of *Euryarchaeota* was found in both purple-colored samples, accounting for 57.2% (DPMM) and 93.2% (DPME) of total sequence reads. The remaining bacterial 16S rRNA gene sequences were affiliated with *Bacteroidetes*, *Proteobacteria* and *Firmicutes* phyla and their relative abundance in DPMM was higher than in DPME (Additional file 2: Figure S2). The archaeal community in DPMM was mainly dominated by 16 s rRNA gene sequences closely related to hydrogenotrophic methanogens comprising of *Methanobacterium* (20.7%), *Methanobrevibacter* (18.8%) and *Methanocorpusculum* (15.3%). *Methanobrevibacter* was found to be most dominant in DPME (85.86%) (Figure 4). While *Methanosarcina* was found to account for 1.1% of DPMM, there was no detection of *Methanosarcina* in DPME. The archaeal communities in both purple-colored samples mainly comprised of hydrogenotrophic methanogens.

## Discussion

Light conditions significantly enhanced power densities (around 8–16%) in the MFCs using mixed and pure cultures of *R. palustris* strains DX-1 and RE-2 respectively (Xing et al. [2009]). In order to better understand electricity generation in MFCs containing purple photosynthetic *Rhodopseudomonas palustris* in mixed culture, microbial communities in DPMM and DPME were investigated using 16S rRNA gene pyrosequencing. As expected, *Rhodopseudomonas* was found to be dominant in both samples, but it was present in much higher abundance in DPME than DPMM; this might be due to the fact that DPME has developed for a longer period of time (2 years) than DPMM (4 weeks). The *Rhodopseudomonas* sp. detected in our study does not provide insight on its capability for electricity generation. The *Rhodopseudomonas* sp. was found to be more closely related with *R. faecalis* and *R. rhenobacensis* which are unknown for their ability to generate electricity, and *R*. palustris ATCC 17001 which does not produce an electric current, but it is less closely related to known electrogenic bacteria *R. palustris* strains DX-1 (Xing et al. [2008]).

Previous studies have shown that *Geobacter*, *Comamonas* and *Pseudomonas* dominated the anode communities of acetate-fed MFCs under light conditions, revealed by PCR/DGGE and 16S rRNA gene library analyses (Xing et al. [2009]). The capacity of electricity generation in acetate-fed MFCs by those genera has also been revealed (Xing et al. [2010]; Pham et al. [2008]; Xing et al. [2008]). However, these genera were found with low abundance in the DPME. This contrast in abundance might indicate that the species structure of the bacterial community differ between the MFC anode biofilm and effluent, whilst also being dependent on inoculum source.

Chemolithotrophic bacterium *Hydrogenophaga*, which consumes hydrogen and carbon dioxide as energy and carbon sources respectively (Willems et al. [1989]). They were found in DPMM but not in DPME. *Geobacter sulfurreducens* has been found in syntrophic cooperation with the *Hydrogenophaga* sp. strain AR20 in acetate-fed MFC (Kimura and Okabe [2013a]). In DPMM, *Hydrogenophaga* might use the hydrogen produced by *Rhodopseudomonas* but not the acetate. Members of chemolithotrophic bacteria were also found in DPME, including *unclassified Ignavibacteriaceae*, *Acinetobacter*, *Desulfovibrio* and *Azospirillum* which are known to be capable of using hydrogen as an electron donor (Wong et al. [1986]; Gross and Simon [2003]; Yong et al. [2002]; Iino et al. [2010]). It has been reported that the growth of *G. sulfurreducens* was more efficient when co-cultured with *Wolinella* and *Desulfovibrio,* which act as hydrogen-consuming partners; with nitrate as the electron acceptor, acetate oxidation was more rapid, resulting in faster growth of *Geobacter sulfurreducens* under low hydrogen partial pressure (Cord-Ruwisch et al. [1998]). The removal of hydrogen by the hydrogen-consuming bacteria in the DPME might have stimulated the growth of *Rhodopseudomonas*. Our results suggested that the growth of *Rhodopseudomonas* was due to the syntrophic cooperation with hydrogen-consuming bacteria in both purple-colored samples.

Anodic hydrogen oxidation by hydrogenotrophic exoelectrogens produces electrical current after complete oxidation of acetate, and a rapid current increase occurs when hydrogen gas is supplied to the reactor (Lee et al. [2009]). However, a previous study showed that electricity generation by hydrogenotrophic exoelectrogens was excluded due to inhibition of nitrogenase-dependent hydrogen production in *Rhodopseudomonas* by NH_4_Cl in the medium (Hillmer and Gest [1977]; Rey et al. [2007]). The presence of hydrogen-oxidizing bacteria in the purple-colored samples suggested that hydrogen production by *Rhodopseudomonas* could not be completely inhibited by NH_4_Cl in the medium (Rey et al. [2007]). Potential hydrogenotrophic exoelectrogens might coexist with *Rhodopseudomonas* in the anode biofilm due to their involvement in electricity generation via hydrogen oxidation. This potential syntrophic interaction between *Rhodopseudomonas* and hydrogen-oxidizing bacteria might be one of the explanations for the high power density obtained from illuminated MFCs.

Bacterial 16S rRNA gene sequences were also detected by the V3-V5 primer set and inconsistent results in the bacterial diversity and the abundance patterns of bacterial community were observed between V1-V3 and V3-V5 data sets. In our previous study (unpublished data), the V3-V5 primer set was also applied for methane-producing biocathodes in MECs, which accounted for 99% of the total sequences belonged to the archaea domain. The PCR amplification of the V3–V5 region of 16S rRNA can be biased depending on the activity of the archaeal population present in the samples. The differences in portions of archaeal sequences between two purple-colored samples suggest that the DPME could have a more active archaeal population than DPMM.

Archaeal community in MFC reactors enriched with *Rhodopseudomonas* sp. has not been reported yet. The predominant hydrogenotrophic methanogens were found in both purple-colored samples, but not acetoclastic methanogens despite the MFC medium containing high concentration of acetate. Under anaerobic conditions, methanogenic archaea often partner with heterotrophic H_2_-producing bacteria which catalyze oxidation of a variety of organic compounds (fatty acids, alcohols, and aromatic compounds). The methanogens utilize the H_2_ produced by these heterotrophic bacteria during methanogenesis while the heterotrophic bacteria benefit from the methanogens, which play a role in the removal of excess hydrogen that would inhibit their growth (McInerney et al. [2008]; Sakai et al. [2009]). Hydrogenotrophic methanogenesis by *Methanobrevibacter* in DPME might be relying on the predominance of *Rhodopseudomonas* for interspecies H_2_ transfer. However, it seems likely that CO_2_ derived from *Rhodopseudomonas* is limited as a major source of carbon for methane generation due to its known role in being recycled into biomass (McKinlay and Harwood [2010]). Hydrogen generation by *Rhodopseudomonas* in both purple-colored samples containing acetate could share similar environmental condition with hydrogen-producing MEC reactors fed with acetate, where hydrogenotrophic methanogens have emerged as the most active methanogens (Lu et al. [2012a]).

In general, few acetoclastic methanogens are present in acetate fed-MFCs due to their inhibition in growth as a result of air exposure and outcompetition for organic substrates by facultative anaerobes and exoelectrogens (Chae et al. [2010]; Dar et al. [2008]). In addition, hydrogen inhibition of growth and acetate metabolism in *Methanosarcina* species has been reported (Ahring et al. [1991]; Ferguson and Mah [1983]). Minor acetoclastic methanogen (*Methanosarcina*) present in the DPMM and the non-detection of *Methanosarcina* in the DPME might be indication of the robust inhibition by hydrogen produced by *Rhodopseudomonas* and its acetate competition.

In conclusion, although the electric generation ability of *Rhodopseudomonas* detected in our study is unknown, putative hydrogenotrophic exoelectrogens existing in MFC reactors having purple-colored effluents might contribute to increasing power density. For future studies the above observations are needed to investigate how known-exoelectrogenic phototrophic bacteria can be applied to efficiently generate electricity via syntrophic relationships with the hydrogenotrophic exoelectrogens and methanogens in the acetate-fed MFC under light conditions.

## Competing interests

The authors declare that they have no competing interests.

## Additional files

## Supplementary Material

Additional file 1: Figure S1. Rarefaction curves based on the V3-V5 primer set with cut-off threshold of 97% similarity in DPMM and DPME. Click here for file

Additional file 2: Figure S2. Microbial community distribution based on the V3-V5 primer set in DPMM and DPME. Click here for file
