# Supplementary figures and images for: Microbial community in microbial fuel cell (MFC) medium and effluent enriched with purple photosynthetic bacterium (Rhodopseudomonas sp.)
Source: AMB Express. 2014 Apr 1;4:22. doi: 10.1186/s13568-014-0022-2 (PMC4052673; doi:10.1186/s13568-014-0022-2)

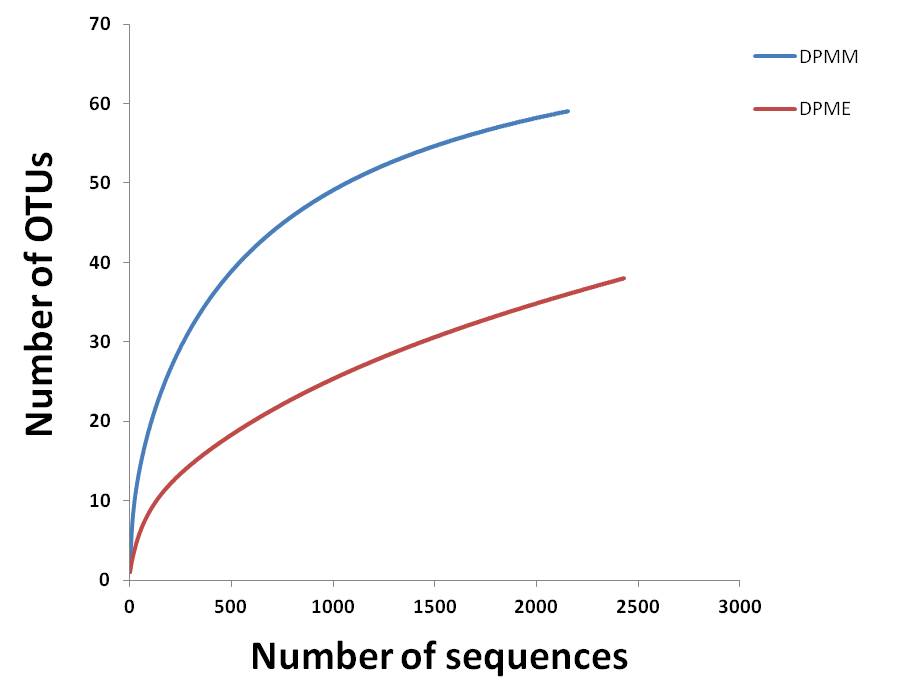

Supplement: Additional file 1: Figure S1. — Rarefaction curves based on the V3-V5 primer set with cut-off threshold of 97% similarity in DPMM and DPME. [file s13568-014-0022-2-S1.jpg]

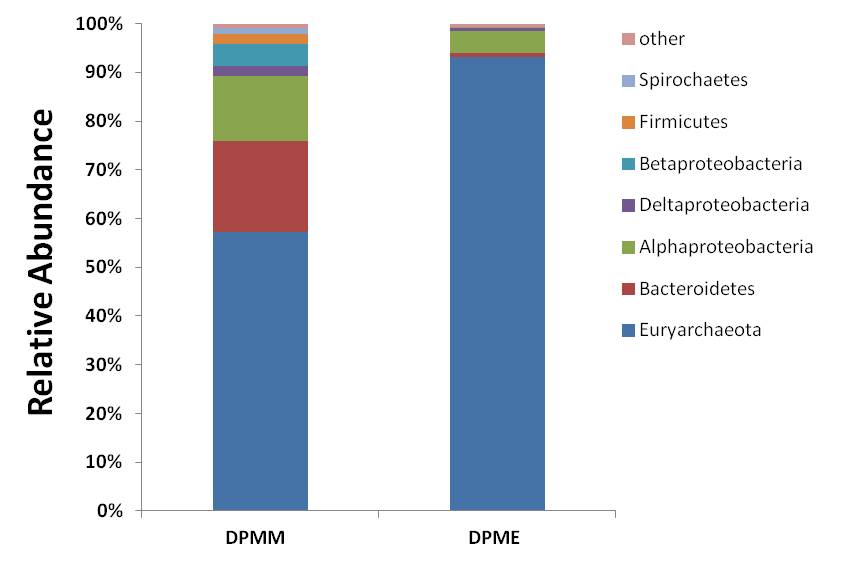

Supplement: Additional file 2: Figure S2. — Microbial community distribution based on the V3-V5 primer set in DPMM and DPME. [file s13568-014-0022-2-S2.jpg]
